# Contributions of substitutions and indels to the structural variations in ancient protein superfamilies

**DOI:** 10.1186/s12864-018-5178-8

**Published:** 2018-10-24

**Authors:** Zheng Zhang, Jinlan Wang, Ya Gong, Yuezhong Li

**Affiliations:** 1 State Key Laboratory of Microbial Technology, Institute of Microbial Technology, Shandong University, Qingdao, 266237 China; 2 Physical Examination Office of Shandong Province, Health and Family Planning Commission of Shandong Province, Jinan, 250014 China

**Keywords:** Indel, Substitution, Genome, Structural variations, Ancient protein superfamilies

## Abstract

**Background:**

Quantitative evaluation of protein structural evolution is important for our understanding of protein biological functions and their evolutionary adaptation, and is useful in guiding protein engineering. However, compared to the models for sequence evolution, the quantitative models for protein structural evolution received less attention. Ancient protein superfamilies are often considered versatile, allowing genetic and functional diversifications during long-term evolution. In this study, we investigated the quantitative impacts of sequence variations on the structural evolution of homologues in 68 ancient protein superfamilies that exist widely in sequenced eukaryotic, bacterial and archaeal genomes.

**Results:**

We found that the accumulated structural variations within ancient superfamilies could be explained largely by a bilinear model that simultaneously considers amino acid substitution and insertion/deletion (indel). Both substitutions and indels are essential for explaining the structural variations within ancient superfamilies. For those ancient superfamilies with high bilinear multiple correlation coefficients, the influence of each unit of substitution or indel on structural variations is almost constant within each superfamily, but varies greatly among different superfamilies. The influence of each unit indel on structural variations is always larger than that of each unit substitution within each superfamily, but the accumulated contributions of indels to structural variations are lower than those of substitutions in most superfamilies. The total contributions of sequence indels and substitutions (46% and 54%, respectively) to the structural variations that result from sequence variations are slightly different in ancient superfamilies.

**Conclusions:**

Structural variations within ancient protein superfamilies accumulated under the significantly bilinear influence of amino acid substitutions and indels in sequences. Both substitutions and indels are essential for explaining the structural variations within ancient superfamilies. For those structural variations resulting from sequence variations, the total contribution of indels is slightly lower than that of amino acid substitutions. The regular clock exists not only in protein sequences, but also probably in protein structures.

**Electronic supplementary material:**

The online version of this article (10.1186/s12864-018-5178-8) contains supplementary material, which is available to authorized users.

## Background

Protein structural evolution is influenced by sequence variations. For example, an early study indicated that, along with the percentage increase of mutated residues within a sequence, the root mean square deviation (RMSD) between the positions of the Cα atoms of two proteins exponentially increased [1]. Subsequent studies also showed that protein structural changes were directly related to sequence divergences [2–9]. However, compared to those for sequence evolution, the quantitative models for protein structural evolution have been less well characterized.

Insertions/deletions (indels) and substitutions are the two most common types of sequence variations. Previous studies showed that indels play important roles in driving divergence of duplicated genes [10–12] and genomic evolution [13, 14]. Several statistical models based on sequence analysis have been established for evaluating the impact of indels on structural changes [15–17]. Substitutions and indels affect the organismal adaptations by providing diverse protein properties [18, 19]. In the case of protein structure, substitutions and indels are regarded as the main factors causing the folding changes between protein homologues [20, 21]. For example, our previous studies show that indels within a protein domain caused the structural shifts of their flanking regions [22]. The structural evolution in protein families is thought to arise from the combined influence of substitutions and indels to a great extent [23].

Ancient protein superfamilies are considered to be versatile during evolution, having produced many genetic and functional variations [24], and thus provide good materials for analysing the structural variations across an extended evolutionary timescale. In this study, we investigated the correlation between protein sequence variations and structure variations in 68 ancient protein superfamilies that are widely present in the sequenced genomes of eukaryotes, bacteria and archaea. We divided the sequence variations into amino acid substitutions and indels and estimated their respective quantitative contributions to the accumulated structural variations within ancient superfamilies.

## Results

### Selection of ancient protein superfamilies in sequenced genomes

The SCOP (Structural Classification of Proteins) superfamilies were assigned to all the proteins encoded in the genome with hidden Markov models to scan protein sequences for 383 model eukaryotes, 1062 model bacteria and 114 model archaea whose genomes have been completely sequenced [25, 26]. Based on the superfamily assignment information, we selected those protein superfamilies existing simultaneously in more than 90% of model eukaryotes, 90% of model bacteria and 90% of model archaea for our analysis. They are thought to belong to the most ancient protein superfamilies and probably existed in the last universal common ancestor (LUCA) [24]. The protein structure data employed in this study were obtained from the ASTRAL95 non-redundant database, in which the sequence identity between any two structures is less than 95% [27]. To ensure statistical reliability, we selected only those superfamilies that contain 20 or more non-redundant structures, as determined by X-ray crystallography.

The 68 ancient superfamilies from the first five SCOP classes were used (Additional file 1: Table S1). Half of these superfamilies are derived from the α/β class, and these proteins are mainly composed of parallel beta sheets (beta-alpha-beta units). Twenty-one superfamilies belong to the α + β class, and these proteins are mainly composed of antiparallel beta sheets (segregated alpha and beta regions). Eight superfamilies are from the “all beta” class, four are from the “all alpha” class, and one is from the multi-domain protein class (α&β). These 68 ancient superfamilies are derived from 60 different folding patterns. Among them, the “TIM beta/alpha-barrel” fold includes six ancient superfamilies, and the “adenine nucleotide alpha hydrolase-like” fold, the “ribonuclease H-like motif” fold, and the “ferredoxin-like” fold contain two ancient superfamilies, respectively. Each of the remaining folds includes only one ancient superfamily.

### Definitions of sequence and structural similarities

The homologous non-redundant domains within each of the ancient SCOP superfamilies were pairwise aligned by the PDBeFold online service [28], yielding 230,133 structure-based sequence alignments of 3711 non-redundant domains from the 68 superfamilies. Of the total alignments, 96.8% were pairs of distantly related protein homologues with a sequence identity lower than 30%. While considering the statistical significances of these alignments, there were 90,816 alignments with P-scores greater than zero, of which the alignments with sequence identity lower than 30% accounted for 91.9%.

We defined two groups of variables for the analysis of sequence-structure correlation in the ancient superfamilies according to these alignments (see methods for details). For Group 1, the structural similarity between two homologous proteins was measured by the RMSD. To estimate the sequence similarity between two homologous proteins, the percent sequence non-identity (PNI) and standardized number of gaps (SNG) parameters were employed. PNI represents the ratio of substituted sites within aligned sequences, and SNG represents the density of gaps that are the potentially non-aligned regions arising from the occurrence of indels. The definitions of Group 1 variables are similar to those in our previous analysis [23].

In addition to Group 1, we further defined a new group of variables (Group 2) for the analysis of those distantly related homologues. For Group 2, structural similarity was quantified by the Z-score of the structural alignment, a variable that measures the statistical significance of a match in terms of Gaussian statistics. The sequence similarity in Group 2 was characterized by the percent sequence non-similarity (PNS) and the length-weighted standardized number of gaps (LSNG). PNS represents the ratio of non-similar substitutions within aligned regions, in which the non-similar substitutions were defined as those amino acid substitutions whose scores in the BLOSUM45 similarity matrix were zero or negative values. LSNG was obtained by weighting SNG according to the lengths of different gaps because of the expectations that longer gap lengths would have greater influences on structure.

**Fig. 1:**
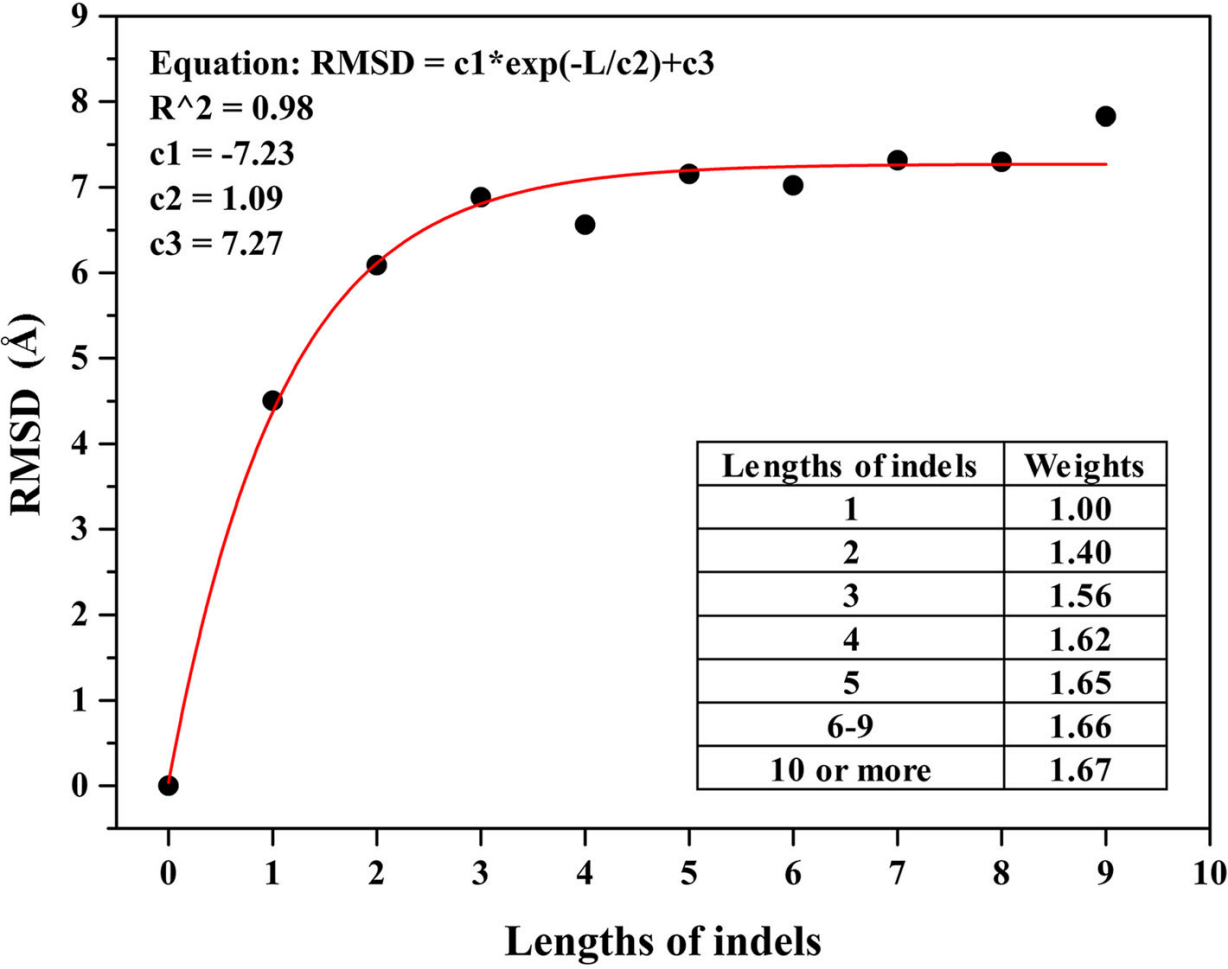
Varying degrees of influence of different length indels on the structural shift of their flanking regions**.** The data derive from Indel Flanking Region Database. Each data point in the figure includes more than 1000 samples. The flanking region contains respective ten amino acid sites nearest to indels within each side of an indel. The degrees of structural shift of the flanking regions (expressed as RMSD) increase with the lengths of their indels (L), the shift can be well fitted by a first-order exponential decay (increasing form) model that could be represented as follows: RMSD = c1*exp.(−L/c2) + c3. In this equation, c1, c2, and c3 are empirical parameters. According to this relationship, the approximate weight values of other length indels can be calculated based on the indels with the length of one amino acid residue. The weight value represents the multiples of total structural shifts of flanking regions for indels with the length of L relative to that of the indels with the length of one amino acid residue

We analysed the influence of different lengths of indels on the structural shift of their flanking regions by using data from the indel flanking region database (IndelFR) [29]. The results showed that the degree of structural shifts of the flanking regions (expressed as the RMSD) increased with the length of their indels (L). The shift was well fitted by an asymptotic growth curve (Fig. 1). That is, with the increase of indel length, their influence on protein structure rises quickly first and then flattens out to some limiting value. According to this relationship, the approximate weight values of indels of other lengths can be calculated based on the indels with the length of one amino acid residue.

### Protein sequence-structure correlation within ancient superfamilies

We analysed the sequence-structure correlation between protein homologues within each of the 68 ancient superfamilies using the variables of Group 1 (PNI, SNG versus RMSD) and Group 2 (PNS, LSNG versus Z-score) (Table 1 and Additional file 2: Table S2). Both of the groups were fitted to produce statistically significant bilinear multiple correlation coefficients (R, *p* < 0.001) within each superfamily. In half of the superfamilies, the bilinear multiple correlation coefficients obtained by fitting Group 2 were greater than 0.808, while the corresponding value was 0.740 for Group 1. If only the relatively accurate alignments (P-score > 0) were considered, both of the R values produced by fitting Group 1 and Group 2 were also statistically significant (*p* < 0.001) for each superfamily, and the median R value obtained by fitting Group 1 increased to 0.800. Thus, even for markedly varied members in the most ancient superfamilies, protein structural variations accumulated under a significantly bilinear influence of amino acid substitutions and indels in the sequences.

**Table 1:** Bilinear multiple correlation coefficients of sequence variations and structural changes within 68 ancient superfamilies

Alignments	Variable groups	Bilinear multiple correlation coefficient, R		
		Median	Upper quartile	Lower quartile
All	Group 1	0.740	0.822	0.662
	Group 2	0.808	0.877	0.733
P-score > 0	Group 1	0.800	0.864	0.678
	Group 2	0.821	0.875	0.742

**Fig. 2:**
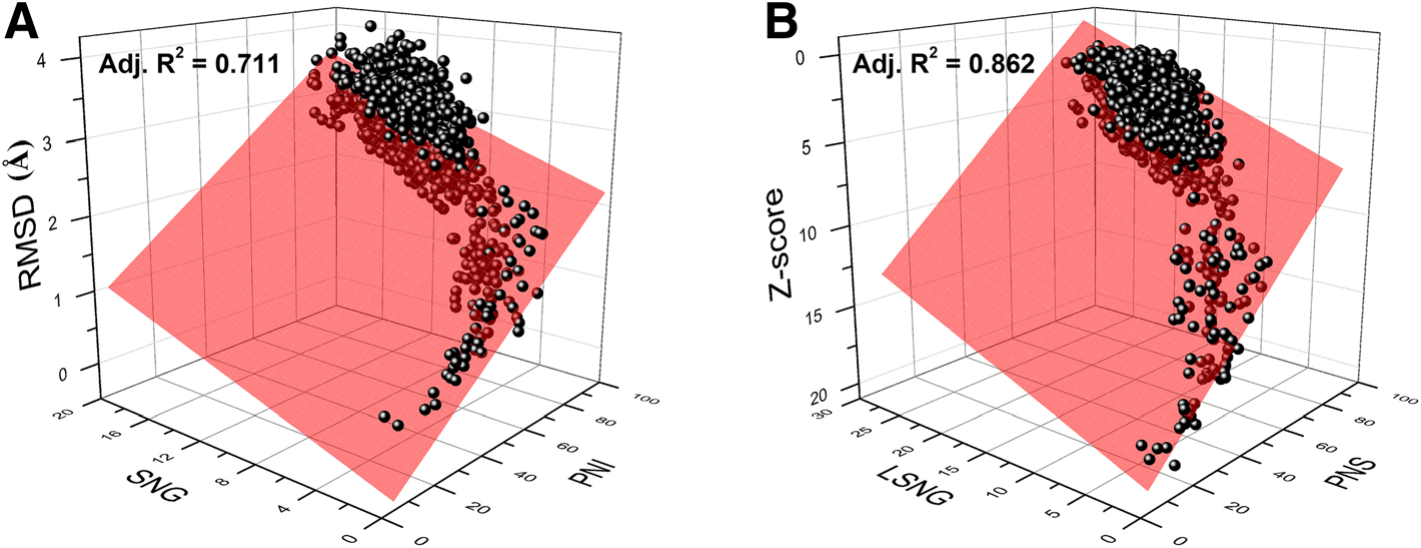
Sequence-structure bilinear correlation within aldolase superfamily. **a** Bilinear correlation of PNI, SNG versus RMSD. **b** Bilinear correlation of PNS, LSNG versus Z-score. The analysis is based on 1596 data

Using the aldolase superfamily (c.1.10) as an example, we displayed the sequence-structure bilinear correlations obtained by fitting Group 1 and Group 2 (Fig. 2). Compared with the result from Group 1, the adjusted R^2^ obtained by fitting Group 2 increased by 21% within this superfamily. Further, the adjusted R^2^ obtained by fitting Group 2 increased by more than 10% compared to that in Group 1 in 54 of the 68 ancient superfamilies (Additional file 3: Table S3). Thus, Group 2 variables were more suitable than Group 1 variables for analysing sequence-structure correlations of distantly related protein homologues.

**Fig. 3:**
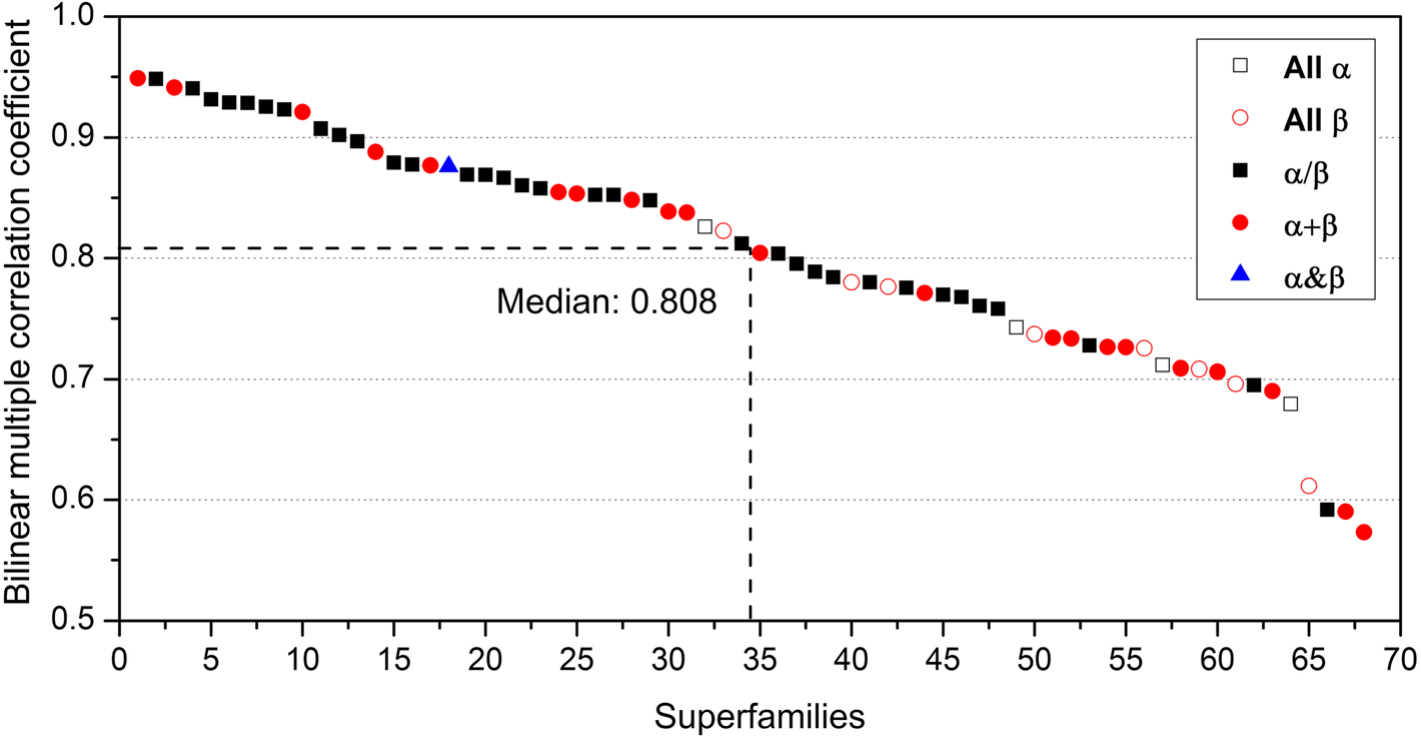
Bilinear multiple correlation coefficients of PNS, LSNG versus Z-score within 68 ancient superfamilies. The SCOP classes to which superfamilies belong are marked by various symbols. Open squares represent all-α class, open circles show all-β class, filled squares represent α/β class, filled circles signify α + β class, and filled triangles represent α&β class

The bilinear multiple correlation coefficients obtained by fitting Group 2 (PNS, LSNG versus Z-score) for all alignments in each of 68 ancient superfamilies are displayed in Fig. 3. The median value is 0.808, which indicated that more than 65% of the structural variations within half of the superfamilies could be explained by the bilinear model. The 68 ancient superfamilies are from the first five SCOP classes (all α, all β, α/β, α + β and α&β), and half of them are from the α/β class. The median of the bilinear multiple correlation coefficients for the superfamilies from the α/β class reached 0.856, which is significantly higher than those from other classes (Table 2). Therefore, the ancient superfamilies from the α/β class are better explained by the bilinear model than those from other classes.

**Table 2:** Comparisons among the bilinear multiple correlation coefficients of the superfamilies from the α/β class and other classes

SCOP class	No. of ancient superfamilies	Bilinear multiple correlation coefficient, R		
		Median	Upper quartile	Lower quartile
α/β	34	0.856	0.903	0.779
others	34	0.757	0.850	0.709

### Substitutions and indels were essential for explaining the structural changes

Indels and substitutions may appear independently or may be correlated. For example, the single-nucleotide substitution rate is higher near sites of indels and decreases as distance from an indel increases [13, 30]. Further, the fixation of indels and substitutions can be due to drift or their ability to modify protein function/structure. The previous study indicated that indels were purged much more intensely than substitutions [31]. To evaluate the necessity of using substitutions and indels to explain structural variations, we further performed partial correlation analysis, adequacy analysis and collinearity diagnostic analysis.

The results showed that, with two exceptions, the partial correlation coefficients within each superfamily were significantly different from zero (*p* < 0.01) between substitution and structural variations (PNI-RMSD, PNS-Z-score) and between indel and structural variation (SNG-RMSD, LSNG-Z-score) (Additional file 4: Table S4). Furthermore, in most of the superfamilies, adequacy analysis indicated that the bilinear model, either using Group 1 or Group 2, significantly improved the linear model when considering only substitutions or indels (Additional file 3: Table S3). The collinearity diagnostic analysis demonstrated that the variance inflation factors (VIFs) were below 5 in all the superfamilies, suggesting that the collinearity between two variables had no significant influence on bilinear fitting. Thus, these analyses indicated that both amino acid substitutions and indels had significant contributions to protein structural changes, and both were essential for explaining the structural changes of protein homologues within these ancient superfamilies.

Collections of pairwise comparisons are not all statistically independent within a family due to the phylogenetic structure of the data. We specifically performed protein comparison analysis across families belonging to a superfamily. Alignments with a sequence identity lower than 30% accounted for 99.9% of all the above alignments. There were 61 superfamilies with two or more families in the 68 total superfamilies that we studied. We analysed sequence-structure correlation between the protein homologues across families within each of these 61 superfamilies using Group 2 (PNS, LSNG versus Z-score). The results showed that Group 2 was fitted to produce a statistically significant bilinear multiple correlation coefficient (R, *p* < 0.001) within each superfamily, but the R value for each superfamily was lower than that in the analysis based on all data, and the median R value was 0.592.

### Contributions of indels to structural variations within the ancient superfamilies

We referred to the absolute values of the two regression coefficients as structural substitution sensitivity (SSS) and structural indel sensitivity (SIDS), which were obtained from regression analysis of bilinear models. For Group 2, SSS represents the structural changes arising from each unit of non-similar substitution sites, while SIDS represents the structural changes caused by each unit of indels with the standard length. In the 48 ancient superfamilies with high bilinear multiple correlation coefficients (*R* > 0.75) obtained by fitting Group 2, although both SSS and SIDS exhibited obvious differences among these superfamilies, SIDS was always higher than SSS within each superfamily (Additional file 4: Table S4). The SIDS/SSS values ranged from 1.02 (c.55.1) to 4.99 (b.38.1). These results suggested that the structural mutation sensitivity varied among different ancient superfamilies and probably reflected the idea that different topological structures have different constraints on sequence variation [32].

**Fig. 4:**
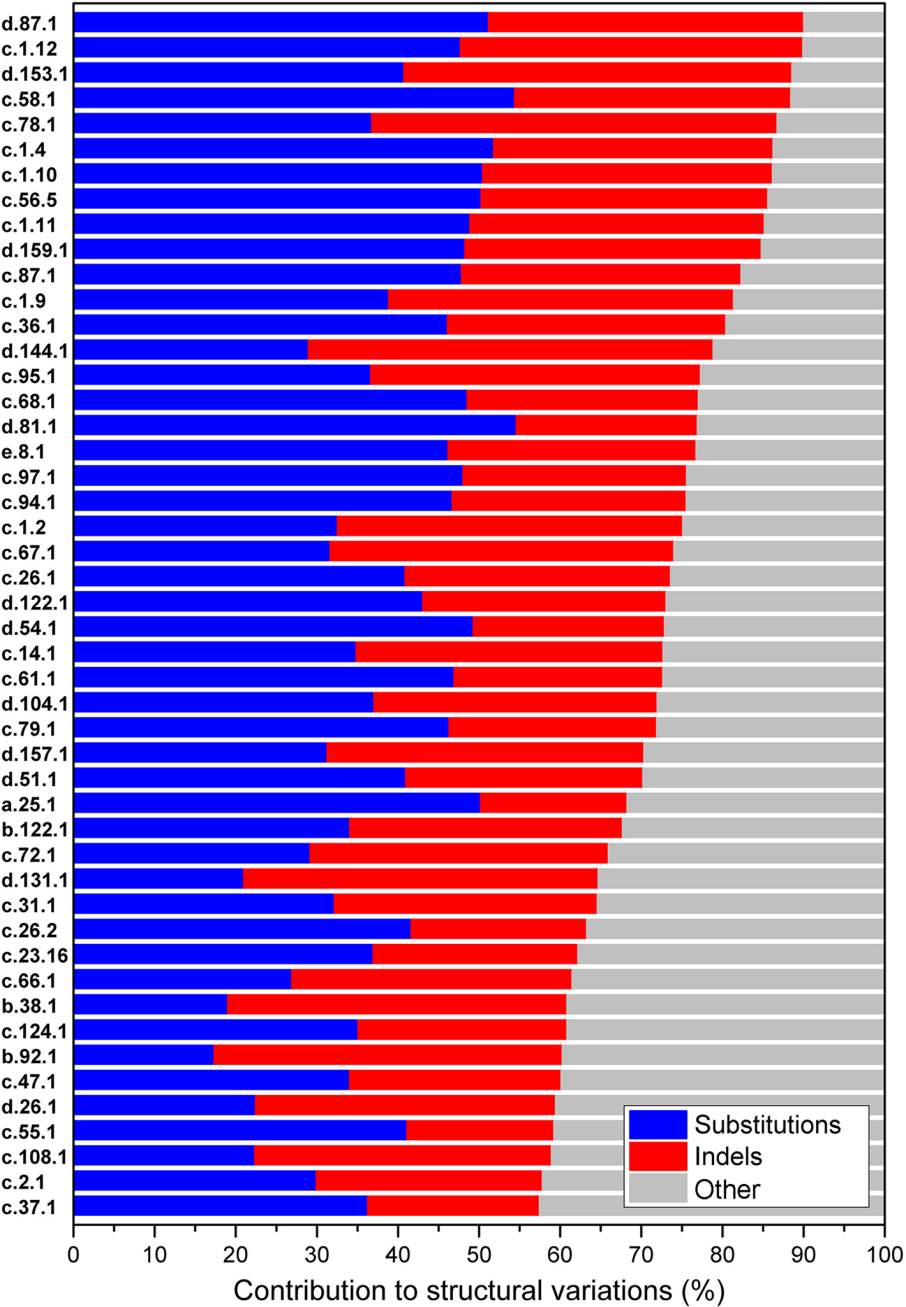
Contributions of amino acid substitutions and indels to structural variations within the ancient superfamilies. It was displayed within 48 superfamilies with high bilinear multiple correlation coefficients (*R* > 0.75) of PNS, LSNG versus Z-score. The identifiers of the superfamilies are on the left. The lengths of the three regions from left to right within each line successively represent the contributions of amino acid substitutions, indels and other factors to structural variations within each superfamily

Further, we calculated the contributions of amino acid substitutions and indels to the accumulation of structural variations by using the product measure [33]. The sum of all the independent variable contributions was equal to the coefficient of determination (R^2^) (Additional file 5: Table S5). The contributions of amino acid substitutions and indels to structural variations are displayed for the 48 ancient superfamilies with high bilinear multiple correlation coefficients (*R* > 0.75) obtained by fitting Group 2 (Fig. 4). The ratio between the contributions of two independent variables reflects their relative importance. Our analyses showed that compared to substitutions, indels had more important contributions to structural variations in 17 superfamilies. However, considering the total structural variations that can be explained by sequence variations within the 48 superfamilies, the total contributions of indels was 46%, which was slightly lower than that of substitutions (54%). These results again indicated that structural variations within ancient protein superfamilies arose from the combined contributions of amino acid substitutions and indels.

## Discussion

In this study, we investigated the quantitative impacts of sequence variations on the structural evolution of homologues in 68 ancient protein superfamilies. Structural variations within ancient protein superfamilies accumulated under the significantly bilinear influence of amino acid substitutions and indels in sequences. It is noteworthy that the bilinear multiple correlation coefficients of four specific superfamilies, including nucleic acid-binding proteins (b.40.4), FAD/NAD(P)-binding domain (c.3.1), CBS-domain pair (d.37.1) and 4Fe-4S ferredoxins (d.58.1), are obviously lower than those of the other superfamilies (Fig. 3). We inferred that the protein stability in these four superfamilies is probably largely influenced by the ligands they bind, thus resulting in weak correlations between sequence and structure.

In addition, for correcting the multiple substitutions per site, we also used the Poisson correction distance (PC) to measure sequence divergences (Additional file 2: Table S2). All the R values obtained by fitting corrected Group 1 variables (PC, SNG versus RMSD) are statistically significant (*p* < 0.001) for all the alignments within each superfamily. However, the median R values obtained by fitting corrected Group 1 is 0.745 that is only slightly better than the corresponding value (0.740) by fitting Group 1. Thus, the multiple substitutions at the same site may not influence the structural variations by means of accumulated pattern.

## Conclusions

Structural variations within the ancient protein superfamilies were accumulated under the significantly bilinear influence of amino acid substitutions and indels in the sequences. Both the amino acid substitutions and indels are essential to explain the structural variations within the ancient superfamilies. For those structural variations resulted from the sequence variations, the total contributions of indels is approximately 46%, slightly lower than that of amino acid substitutions (54%). Thus, the significantly bilinear correlation between sequence variations and structural changes indicates that the regular clock exists not only in protein sequences, but also probably in protein structures.

## Methods

### Selection of the ancient superfamilies and acquisition of structural datasets

The data for homologous protein superfamilies were retrieved from the SCOP structural classification database [25]. Structural data came from the ASTRAL95 non-redundant structure database, with less than 95% identity to any pair of its sequences [27]. By using the annotations of the SUPERFAMILY database, we analysed 383 completely sequenced model eukaryotes, 1062 completely sequenced model bacteria and 114 completely sequenced model archaea [26]. We selected those superfamilies existing simultaneously in over 90% of model eukaryotes, 90% of model bacteria and 90% of model archaea. These were considered to be the most ancient protein superfamilies. Then, our analysis was limited to the first five SCOP classes, including all alpha proteins (all α), all beta proteins (all β), alpha and beta proteins (α/β, beta-alpha-beta units), alpha and beta proteins (α + β, segregated alpha and beta regions), and multi-domain proteins (α&β). The superfamilies that contain 20 or more non-redundant structures determined by X-ray crystallography were selected for further analysis. Ultimately, 68 ancient superfamilies with 3711 non-redundant domains were studied (Additional file 1: Table S1).

### Measuring structural and sequence similarities

Homologous non-redundant domains within each ancient superfamily were pairwise aligned by the PDBeFold online service [28]. The software, based on identification of residues occupying “equivalent” geometrical positions, provides pairwise and multiple comparison, C-α alignment and examination similarity. The P-score represents the negative logarithm of the *P*-value, and it is able to measure the quality of alignment. The P-value is calculated according to the RMSD, number of aligned residues, number of gaps, number of matched secondary structure elements (SSE) and the SSE match score. The higher the P-score, the more statistically significant the alignment is.

Within 68 superfamilies, 230,133 structure-based sequence alignments were obtained. While considering the statistical significances of these alignments, we also specifically analysed the alignments with P-scores greater than zero. Alignments containing less than 50 aligned residues were removed. According to these alignment results, we defined two groups of variables to analyse the sequence-structure correlation.

For Group 1, structural similarity between two homologous proteins was measured by the RMSD, and sequence similarity was quantified by percent sequence non-identity (PNI) and standardized number of gaps (SNG). These definitions are similar to those in previous research [23]. The RMSD was directly acquired from the alignment results, while PNI and SNG were calculated as follows:

1 $$ PNI=\left(1-{N}_{iden}/{N}_{algn}\right)\ast 100 $$

2 $$ SNG=100\ast {N}_{gap}/{N}_{algn} $$

where *N*_*iden*_ is the number of identical sites within each alignment, *N*_*algn*_ is the number of amino acid residues within each alignment, and *N*_*gap*_ is the total number of gaps within each alignment irrespective of the length of each individual gap.

For Group 2, the structural similarity was quantified by the Z-scores of structural alignments, and the sequence similarity was measured by the percent sequence non-similarity (PNS) and length-weighted standardized number of gaps (LSNG). The Z-score was directly acquired from the alignment results, while both PNS and LSNG were calculated as follows:

3 $$ PNS=100\ast {N}_{ns}/{N}_{algn} $$

4 $$ LSNG=\frac{100}{N_{algn}}\ast \sum \limits_{i=1}^{\infty }{N}_i{a}_i $$

where *N*_*ns*_ is the number of non-similar amino acid sites within each alignment, *N*_*algn*_ is the number of amino acid residues within each alignment, *N*_*i*_ is the total number of gaps with the length of *i* sites within each alignment, and *a*_*i*_ is the weight value of the gaps with the length of *i* sites. The non-similar substitutions mentioned above were defined as those substitutions with BLOSUM45 similarity matrix scores that were zero or negative. The weighted values of different length gaps were estimated by analysing the data from the indel flanking region database (Fig. 1) [22, 29].

### Statistical analysis

Statistical analysis was performed by SigmaStat 3.5. We performed correlation and regression analysis within each ancient superfamily. For each superfamily, we calculated the bilinear multiple correlation coefficient, the partial correlation coefficient between substitutions and structural variations (PNI-RMSD, PNS-Z-score) and the corresponding value between indels and structural variations (SNG-RMSD, LSNG-Z-score). Then, the *p*-values were calculated under the null hypothesis that the correlation coefficients equal zero. The bilinear models constructed by using Group 1 or Group 2 variables are displayed as follows:

5 $$ RMSD={b}_0+{b}_1 PNI+{b}_2 SNG+u $$

6 $$ Z- score={b}_0+{b}_1 PNS+{b}_2 LSNG+u $$

where *b*_0_ is the constant term, both *b*_1_ and *b*_2_ are regression coefficients, and *u* is the error term. We termed *b*_1_ the structural substitution sensitivity (SSS) and considered *b*_2_ the structural indel sensitivity (SIDS).

Adequacy (*r*^2^) analysis was used to quantify the improvements obtained using the bilinear model compared to the linear model that considered only substitution or indels. The adequacy of bilinear fitting versus linear fitting is defined as:

7 $$ {r}^2={adjR^2}_{bilinear}/{adjR}_{linear}^2 $$

where the *adjR*^2^ is the coefficient of determination adjusted according to the number of independent variables and the number of data points in the linear or bilinear fitting. Each coefficient of determination (*R*^2^) was adjusted by:

8 $$ {adjR}^2=1-\left(1-{R}^2\right)\frac{n-1}{n-k-1} $$

where *n* is the number of data points and *k* is the number of independent variables in the fitting.

Within the bilinear model, the contribution of each independent variable to the dependent variable was calculated separately by the product measure [33]. The contribution of *x*_*i*_ to *y* was defined as *C*_*i*_ as follows:

9 $$ {C}_i={\beta}_i{r}_i $$

where *β*_*i*_ is the standardized regression coefficients of *x*_*i*_, and *r*_*i*_ is the simple correlation coefficient between *x*_*i*_ and *y*.

## Additional files

Additional file 1: **Table S1.** Information of 68 ancient superfamilies. (DOCX 22 kb)

Additional file 2: **Table S2.** Bilinear multiple correlation coefficient of sequence variations and structure changes within each of 68 ancient superfamilies. (DOCX 23 kb)

Additional file 3: **Table S3.** Results of adequacy analysis. (DOCX 22 kb)

Additional file 4: **Table S4.** Fitting results of bilinear model within each of 68 ancient superfamilies. (DOCX 24 kb)

Additional file 5: **Table S5.** Accumulated contributions of substitutions and indels to structural variations within the ancient superfamilies. (DOCX 17 kb)

## Abbreviations

Indel: Insertion/deletion
IndelFR: Indel flanking region database
LSNG: Length-weighted standardized number of gaps
LUCA: Last Universal Common Ancestor
PNI: Percent sequence non-identity
PNS: Percent sequence non-similarity
RMSD: Root mean square deviation
SCOP: Structural Classification of Proteins
SIDS: Structural indel sensitivity
SNG: Standardized number of gaps
SSS: Structural substitution sensitivity
VIF: Variance inflation factors
