# Supplementary material for: Contributions of substitutions and indels to the structural variations in ancient protein superfamilies
Source: BMC Genomics. 2018 Oct 24;19:771. doi: 10.1186/s12864-018-5178-8 (PMC6201574; doi:10.1186/s12864-018-5178-8)
Supplement: Supplementary file 1 — Table S1. Information of 68 ancient superfamilies. (DOCX 22 kb) [file 12864_2018_5178_MOESM1_ESM.docx]

**Table S1. Information of 68 ancient superfamilies.**

| No. | SCOP superfamily name^a^ | Code | A%^b^ | B%^c^ | E%^d^ | ASTRAL95^e^ |
| --- | --- | --- | --- | --- | --- | --- |
| 1 | ["Winged helix" DNA-binding domain](http://scop.mrc-lmb.cam.ac.uk/scop/data/scop.b.b.j.e.html) | a.4.5 | 100 | 99.3 | 99.7 | 183 |
| 2 | [Ferritin-like](http://scop.mrc-lmb.cam.ac.uk/scop/data/scop.b.b.di.b.html) | a.25.1 | 99.1 | 98.9 | 99.5 | 61 |
| 3 | [lambda repressor-like DNA-binding domains](http://scop.mrc-lmb.cam.ac.uk/scop/data/scop.b.b.fa.b.html) | a.35.1 | 100 | 95.0 | 99.5 | 43 |
| 4 | [6-phosphogluconate dehydrogenase C-terminal domain-like](http://scop.mrc-lmb.cam.ac.uk/scop/data/scop.b.b.caa.b.html) | a.100.1 | 100 | 99.3 | 99.2 | 23 |
| 5 | [PDZ domain-like](http://scop.mrc-lmb.cam.ac.uk/scop/data/scop.b.c.ge.b.html) | b.36.1 | 99.1 | 96.0 | 99.0 | 88 |
| 6 | [Sm-like ribonucleoproteins](http://scop.mrc-lmb.cam.ac.uk/scop/data/scop.b.c.gg.b.html) | b.38.1 | 100 | 93.3 | 99.7 | 22 |
| 7 | [Nucleic acid-binding proteins](http://scop.mrc-lmb.cam.ac.uk/scop/data/scop.b.c.hb.h.html) | b.40.4 | 100 | 100 | 100 | 110 |
| 8 | [Translation proteins](http://scop.mrc-lmb.cam.ac.uk/scop/data/scop.b.c.he.e.html) | b.43.3 | 100 | 100 | 100 | 23 |
| 9 | [FMN-binding split barrel](http://scop.mrc-lmb.cam.ac.uk/scop/data/scop.b.c.hg.b.html) | b.45.1 | 99.1 | 90.9 | 95.8 | 27 |
| 10 | [RmlC-like cupins](http://scop.mrc-lmb.cam.ac.uk/scop/data/scop.b.c.bdi.b.html) | b.82.1 | 96.5 | 92.7 | 99.5 | 59 |
| 11 | [Composite domain of metallo-dependent hydrolases](http://scop.mrc-lmb.cam.ac.uk/scop/data/scop.b.c.bgd.b.html) | b.92.1 | 99.1 | 93.0 | 94.8 | 21 |
| 12 | [PUA domain-like](http://scop.mrc-lmb.cam.ac.uk/scop/data/scop.b.c.bgj.b.html) | b.122.1 | 100 | 99.0 | 99.7 | 33 |
| 13 | [Ribulose-phoshate binding barrel](http://scop.mrc-lmb.cam.ac.uk/scop/data/scop.b.d.b.c.html) | c.1.2 | 99.1 | 97.4 | 98.7 | 39 |
| 14 | [FMN-linked oxidoreductases](http://scop.mrc-lmb.cam.ac.uk/scop/data/scop.b.d.b.f.A.html) | c.1.4 | 99.1 | 97.7 | 99.7 | 28 |
| 15 | [Metallo-dependent hydrolases](http://scop.mrc-lmb.cam.ac.uk/scop/data/scop.b.d.b.ba.html) | c.1.9 | 100 | 99.5 | 99.7 | 39 |
| 16 | [Aldolase](http://scop.mrc-lmb.cam.ac.uk/scop/data/scop.b.d.b.bb.html) | c.1.10 | 99.1 | 99.9 | 99.7 | 57 |
| 17 | [Enolase C-terminal domain-like](http://scop.mrc-lmb.cam.ac.uk/scop/data/scop.b.d.b.bc.html) | c.1.11 | 99.1 | 97.3 | 99.7 | 27 |
| 18 | [Phosphoenolpyruvate/pyruvate domain](http://scop.mrc-lmb.cam.ac.uk/scop/data/scop.b.d.b.bd.html) | c.1.12 | 99.1 | 99.3 | 99.7 | 23 |
| 19 | [NAD(P)-binding Rossmann-fold domains](http://scop.mrc-lmb.cam.ac.uk/scop/data/scop.b.d.c.b.html) | c.2.1 | 100 | 100 | 99.7 | 302 |
| 20 | [FAD/NAD(P)-binding domain](http://scop.mrc-lmb.cam.ac.uk/scop/data/scop.b.d.d.b.html) | c.3.1 | 100 | 100 | 99.7 | 94 |
| 21 | [ClpP/crotonase](http://scop.mrc-lmb.cam.ac.uk/scop/data/scop.b.d.be.b.html) | c.14.1 | 94.7 | 97.7 | 99.5 | 39 |
| 22 | [Class I glutamine amidotransferase-like](http://scop.mrc-lmb.cam.ac.uk/scop/data/scop.b.d.ch.bf.html) | c.23.16 | 100 | 100 | 100 | 35 |
| 23 | [Nucleotidylyl transferase](http://scop.mrc-lmb.cam.ac.uk/scop/data/scop.b.d.da.b.html) | c.26.1 | 100 | 100 | 99.7 | 43 |
| 24 | [Adenine nucleotide alpha hydrolases-like](http://scop.mrc-lmb.cam.ac.uk/scop/data/scop.b.d.da.c.html) | c.26.2 | 100 | 100 | 100 | 31 |
| 25 | [DHS-like NAD/FAD-binding domain](http://scop.mrc-lmb.cam.ac.uk/scop/data/scop.b.d.dj.b.html) | c.31.1 | 100 | 94.1 | 99.7 | 25 |
| 26 | [Thiamin diphosphate-binding fold (THDP-binding)](http://scop.mrc-lmb.cam.ac.uk/scop/data/scop.b.d.fa.b.html) | c.36.1 | 99.1 | 98.8 | 99.7 | 46 |
| 27 | [P-loop containing nucleoside triphosphate hydrolases](http://scop.mrc-lmb.cam.ac.uk/scop/data/scop.b.d.fb.b.html) | c.37.1 | 100 | 100 | 100 | 409 |
| 28 | [Thioredoxin-like](http://scop.mrc-lmb.cam.ac.uk/scop/data/scop.b.d.gi.b.html) | c.47.1 | 99.1 | 99.7 | 99.7 | 176 |
| 29 | [Restriction endonuclease-like](http://scop.mrc-lmb.cam.ac.uk/scop/data/scop.b.d.he.b.html) | c.52.1 | 100 | 98.5 | 99.5 | 40 |
| 30 | [Actin-like ATPase domain](http://scop.mrc-lmb.cam.ac.uk/scop/data/scop.b.d.hh.b.html) | c.55.1 | 100 | 100 | 100 | 97 |
| 31 | [Ribonuclease H-like](http://scop.mrc-lmb.cam.ac.uk/scop/data/scop.b.d.hh.d.html) | c.55.3 | 100 | 100 | 100 | 60 |
| 32 | [Zn-dependent exopeptidases](http://scop.mrc-lmb.cam.ac.uk/scop/data/scop.b.d.hi.f.html) | c.56.5 | 100 | 99.7 | 99.7 | 44 |
| 33 | [Aminoacid dehydrogenase-like, N-terminal domain](http://scop.mrc-lmb.cam.ac.uk/scop/data/scop.b.d.cb.b.html) | c.58.1 | 100 | 99.0 | 99.7 | 23 |
| 34 | [PRTase-like](http://scop.mrc-lmb.cam.ac.uk/scop/data/scop.b.d.ih.b.html) | c.61.1 | 99.1 | 98.9 | 99.7 | 41 |
| 35 | [S-adenosyl-L-methionine-dependent methyltransferases](http://scop.mrc-lmb.cam.ac.uk/scop/data/scop.b.d.je.b.html) | c.66.1 | 100 | 100 | 99.7 | 120 |
| 36 | [PLP-dependent transferases](http://scop.mrc-lmb.cam.ac.uk/scop/data/scop.b.d.jg.b.html) | c.67.1 | 99.1 | 100 | 100 | 89 |
| 37 | [Nucleotide-diphospho-sugar transferases](http://scop.mrc-lmb.cam.ac.uk/scop/data/scop.b.d.jh.b.html) | c.68.1 | 100 | 98.6 | 99.7 | 38 |
| 38 | [Ribokinase-like](http://scop.mrc-lmb.cam.ac.uk/scop/data/scop.b.d.bad.b.html) | c.72.1 | 99.1 | 95.7 | 99.5 | 25 |
| 39 | [Aspartate/ornithine carbamoyltransferase](http://scop.mrc-lmb.cam.ac.uk/scop/data/scop.b.d.bbg.b.A.html) | c.78.1 | 99.1 | 93.7 | 95.8 | 22 |
| 40 | [Tryptophan synthase beta subunit-like PLP-dependent enzymes](http://scop.mrc-lmb.cam.ac.uk/scop/data/scop.b.d.bbh.b.A.html) | c.79.1 | 99.1 | 93.8 | 97.1 | 22 |
| 41 | [UDP-Glycosyltransferase/glycogen phosphorylase](http://scop.mrc-lmb.cam.ac.uk/scop/data/scop.b.d.bch.b.html) | c.87.1 | 100 | 96.5 | 99.7 | 20 |
| 42 | [Periplasmic binding protein-like II](http://scop.mrc-lmb.cam.ac.uk/scop/data/scop.b.d.beb.b.html) | c.94.1 | 100 | 100 | 94.5 | 80 |
| 43 | [Thiolase-like](http://scop.mrc-lmb.cam.ac.uk/scop/data/scop.b.d.bec.b.html) | c.95.1 | 99.1 | 96.2 | 99.2 | 44 |
| 44 | [Cytidine deaminase-like](http://scop.mrc-lmb.cam.ac.uk/scop/data/scop.b.d.beh.b.html) | c.97.1 | 95.6 | 99.3 | 99.7 | 22 |
| 45 | [HAD-like](http://scop.mrc-lmb.cam.ac.uk/scop/data/scop.b.d.ee.b.html) | c.108.1 | 100 | 99.7 | 99.7 | 58 |
| 46 | [NagB/RpiA/CoA transferase-like](http://scop.mrc-lmb.cam.ac.uk/scop/data/scop.b.d.ef.b.html) | c.124.1 | 99.1 | 98.2 | 99.7 | 27 |
| 47 | [Ribosomal protein S5 domain 2-like](http://scop.mrc-lmb.cam.ac.uk/scop/data/scop.b.e.cj.b.html) | d.14.1 | 100 | 100 | 100 | 46 |
| 48 | [FKBP-like](http://scop.mrc-lmb.cam.ac.uk/scop/data/scop.b.e.ha.b.html) | d.26.1 | 100 | 99.8 | 99.7 | 30 |
| 49 | [CBS-domain pair](http://scop.mrc-lmb.cam.ac.uk/scop/data/scop.b.e.jb.b.A.html) | d.37.1 | 100 | 99.3 | 99.5 | 27 |
| 50 | [dsRNA-binding domain-like](http://scop.mrc-lmb.cam.ac.uk/scop/data/scop.b.e.bag.b.html) | d.50.1 | 100 | 100 | 100 | 25 |
| 51 | [Eukaryotic type KH-domain (KH-domain type I)](http://scop.mrc-lmb.cam.ac.uk/scop/data/scop.b.e.bbe.b.A.html) | d.51.1 | 100 | 98.0 | 99.7 | 25 |
| 52 | [Enolase N-terminal domain-like](http://scop.mrc-lmb.cam.ac.uk/scop/data/scop.b.e.bca.b.A.html) | d.54.1 | 98.2 | 97.2 | 99.7 | 27 |
| 53 | [4Fe-4S ferredoxins](http://scop.mrc-lmb.cam.ac.uk/scop/data/scop.b.e.bcj.b.html) | d.58.1 | 100 | 92.7 | 99.7 | 28 |
| 54 | [ACT-like](http://scop.mrc-lmb.cam.ac.uk/scop/data/scop.b.e.bcj.cc.html) | d.58.18 | 100 | 95.3 | 93.2 | 29 |
| 55 | [Glyceraldehyde-3-phosphate dehydrogenase-like, C-terminal domain](http://scop.mrc-lmb.cam.ac.uk/scop/data/scop.b.e.bih.b.html) | d.81.1 | 99.1 | 99.6 | 99.7 | 49 |
| 56 | [FAD/NAD-linked reductases, dimerisation (C-terminal) domain](http://scop.mrc-lmb.cam.ac.uk/scop/data/scop.b.e.bjj.b.A.html) | d.87.1 | 94.7 | 96.4 | 98.7 | 21 |
| 57 | [Class II aaRS and biotin synthetases](http://scop.mrc-lmb.cam.ac.uk/scop/data/scop.b.e.ccj.b.html) | d.104.1 | 100 | 100 | 99.7 | 33 |
| 58 | [Acyl-CoA N-acyltransferases (Nat)](http://scop.mrc-lmb.cam.ac.uk/scop/data/scop.b.e.cdf.b.html) | d.108.1 | 100 | 97.5 | 99.7 | 75 |
| 59 | [Nudix](http://scop.mrc-lmb.cam.ac.uk/scop/data/scop.b.e.ced.b.html) | d.113.1 | 97.4 | 95.7 | 99.7 | 32 |
| 60 | [ATPase domain of HSP90 chaperone/DNA topoisomerase II/histidine kinase](http://scop.mrc-lmb.cam.ac.uk/scop/data/scop.b.e.cff.b.html) | d.122.1 | 100 | 99.8 | 99.7 | 23 |
| 61 | [DNA clamp](http://scop.mrc-lmb.cam.ac.uk/scop/data/scop.b.e.chb.b.html) | d.131.1 | 100 | 99.5 | 99.7 | 24 |
| 62 | [Glutathione synthetase ATP-binding domain-like](http://scop.mrc-lmb.cam.ac.uk/scop/data/scop.b.e.cji.b.html) | d.142.1 | 100 | 97.4 | 99.7 | 24 |
| 63 | [Protein kinase-like (PK-like)](http://scop.mrc-lmb.cam.ac.uk/scop/data/scop.b.e.daa.b.html) | d.144.1 | 100 | 96.0 | 99.7 | 77 |
| 64 | [N-terminal nucleophile aminohydrolases (Ntn hydrolases)](http://scop.mrc-lmb.cam.ac.uk/scop/data/scop.b.e.dba.b.html) | d.153.1 | 100 | 96.3 | 99.7 | 58 |
| 65 | [Metallo-hydrolase/oxidoreductase](http://scop.mrc-lmb.cam.ac.uk/scop/data/scop.b.e.dbe.b.html) | d.157.1 | 100 | 99.4 | 99.7 | 23 |
| 66 | [Metallo-dependent phosphatases](http://scop.mrc-lmb.cam.ac.uk/scop/data/scop.b.e.dbg.b.html) | d.159.1 | 100 | 97.1 | 99.7 | 23 |
| 67 | [Nucleotidyltransferase](http://scop.mrc-lmb.cam.ac.uk/scop/data/scop.b.e.bbg.b.html) | d.218.1 | 100 | 98.2 | 99.7 | 21 |
| 68 | [DNA/RNA polymerases](http://scop.mrc-lmb.cam.ac.uk/scop/data/scop.b.f.bf.b.html) | e.8.1 | 100 | 98.4 | 99.7 | 37 |

^a^ The superfamily information derives from the SCOP database, the superfamily information included in each specie derives from the SUPERFAMILY database.

^b^ The ratio of those model archaea possessing this superfamily in 114 completely sequenced model archaea.

^c^ The ratio of those model bacteria possessing this superfamily in 1062 completely sequenced model bacteria.

^d^ The ratio of those model eukaryotes possessing this superfamily in 383 completely sequenced model eukaryotes.

^e^ The number of ASTRAL95 non-redundant structures within each superfamily.
