# Supplementary material for: Contributions of substitutions and indels to the structural variations in ancient protein superfamilies
Source: BMC Genomics. 2018 Oct 24;19:771. doi: 10.1186/s12864-018-5178-8 (PMC6201574; doi:10.1186/s12864-018-5178-8)
Supplement: Supplementary file 2 — Table S2. Bilinear multiple correlation coefficient of sequence variations and structure changes within each of 68 ancient superfamilies. (DOCX 23 kb) [file 12864_2018_5178_MOESM2_ESM.docx]

**Table S2.** **Bilinear multiple correlation coefficient of sequence variations and structure changes within each of 68 ancient superfamilies.**

| SCOP code | All alignments | | | | Accurate alignments | | |
| --- | --- | --- | --- | --- | --- | --- | --- |
|  | **N^a^** | **R-Group 1^b^** | **R-Group 2^c^** | **R-PC^d^** | **N^e^** | **R-Group 1^f^** | **R-Group 2^g^** |
| a.4.5 | 16653 | 0.600 | 0.679 | 0.600 | 13316 | 0.586 | 0.650 |
| a.25.1 | 1830 | 0.742 | 0.826 | 0.774 | 1145 | 0.752 | 0.818 |
| a.35.1 | 903 | 0.625 | 0.743 | 0.612 | 812 | 0.605 | 0.710 |
| a.100.1 | 253 | 0.523 | 0.711 | 0.492 | 44 | 0.739 | 0.745 |
| b.36.1 | 3828 | 0.669 | 0.696 | 0.674 | 3708 | 0.649 | 0.662 |
| b.38.1 | 231 | 0.744 | 0.780 | 0.737 | 218 | 0.671 | 0.674 |
| b.40.4 | 5953 | 0.496 | 0.612 | 0.497 | 4049 | 0.522 | 0.609 |
| b.43.3 | 253 | 0.717 | 0.725 | 0.730 | 174 | 0.832 | 0.777 |
| b.45.1 | 351 | 0.724 | 0.737 | 0.722 | 277 | 0.656 | 0.697 |
| b.82.1 | 1596 | 0.700 | 0.708 | 0.708 | 679 | 0.658 | 0.704 |
| b.92.1 | 210 | 0.843 | 0.776 | 0.883 | 178 | 0.714 | 0.506 |
| b.122.1 | 528 | 0.693 | 0.823 | 0.689 | 310 | 0.807 | 0.844 |
| c.1.2 | 741 | 0.825 | 0.867 | 0.815 | 629 | 0.837 | 0.883 |
| c.1.4 | 378 | 0.894 | 0.929 | 0.900 | 224 | 0.952 | 0.947 |
| c.1.9 | 741 | 0.842 | 0.902 | 0.858 | 219 | 0.912 | 0.946 |
| c.1.10 | 1596 | 0.844 | 0.928 | 0.848 | 506 | 0.920 | 0.959 |
| c.1.11 | 351 | 0.880 | 0.923 | 0.880 | 274 | 0.878 | 0.908 |
| c.1.12 | 253 | 0.900 | 0.948 | 0.916 | 96 | 0.935 | 0.964 |
| c.2.1 | 45150 | 0.683 | 0.760 | 0.693 | 21802 | 0.732 | 0.764 |
| c.3.1 | 4371 | 0.644 | 0.592 | 0.642 | 2653 | 0.654 | 0.648 |
| c.14.1 | 741 | 0.790 | 0.853 | 0.776 | 437 | 0.753 | 0.851 |
| c.23.16 | 595 | 0.700 | 0.789 | 0.721 | 356 | 0.796 | 0.831 |
| c.26.1 | 903 | 0.742 | 0.858 | 0.751 | 184 | 0.833 | 0.867 |
| c.26.2 | 465 | 0.661 | 0.795 | 0.641 | 214 | 0.699 | 0.798 |
| c.31.1 | 300 | 0.708 | 0.804 | 0.721 | 226 | 0.702 | 0.786 |
| c.36.1 | 1035 | 0.842 | 0.897 | 0.842 | 792 | 0.861 | 0.904 |
| c.37.1 | 81406 | 0.628 | 0.758 | 0.610 | 9997 | 0.811 | 0.836 |
| c.47.1 | 15400 | 0.682 | 0.775 | 0.660 | 8324 | 0.752 | 0.779 |
| c.52.1 | 780 | 0.465 | 0.695 | 0.471 | 33 | 0.865 | 0.909 |
| c.55.1 | 4656 | 0.566 | 0.770 | 0.506 | 1180 | 0.774 | 0.868 |
| c.55.3 | 1770 | 0.564 | 0.728 | 0.575 | 248 | 0.814 | 0.767 |
| c.56.5 | 861 | 0.868 | 0.925 | 0.906 | 367 | 0.900 | 0.924 |
| c.58.1 | 253 | 0.886 | 0.940 | 0.893 | 90 | 0.940 | 0.924 |
| c.61.1 | 820 | 0.732 | 0.852 | 0.728 | 385 | 0.838 | 0.875 |
| c.66.1 | 7140 | 0.688 | 0.784 | 0.706 | 2988 | 0.657 | 0.754 |
| c.67.1 | 3916 | 0.814 | 0.860 | 0.822 | 1422 | 0.887 | 0.890 |
| c.68.1 | 703 | 0.779 | 0.878 | 0.804 | 225 | 0.849 | 0.905 |
| c.72.1 | 300 | 0.737 | 0.812 | 0.749 | 145 | 0.749 | 0.801 |
| c.78.1 | 190 | 0.901 | 0.931 | 0.936 | 113 | 0.905 | 0.873 |
| c.79.1 | 231 | 0.845 | 0.848 | 0.865 | 199 | 0.843 | 0.834 |
| c.87.1 | 190 | 0.796 | 0.907 | 0.805 | 25 | 0.847 | 0.873 |
| c.94.1 | 3160 | 0.786 | 0.869 | 0.782 | 307 | 0.870 | 0.662 |
| c.95.1 | 946 | 0.777 | 0.879 | 0.786 | 603 | 0.858 | 0.906 |
| c.97.1 | 231 | 0.830 | 0.869 | 0.838 | 166 | 0.896 | 0.842 |
| c.108.1 | 1653 | 0.662 | 0.768 | 0.661 | 620 | 0.648 | 0.787 |
| c.124.1 | 325 | 0.770 | 0.780 | 0.764 | 49 | 0.869 | 0.854 |
| d.14.1 | 1035 | 0.505 | 0.706 | 0.465 | 506 | 0.599 | 0.769 |
| d.26.1 | 406 | 0.801 | 0.771 | 0.800 | 334 | 0.821 | 0.799 |
| d.37.1 | 296 | 0.483 | 0.573 | 0.449 | 296 | 0.483 | 0.573 |
| d.50.1 | 300 | 0.610 | 0.726 | 0.653 | 284 | 0.583 | 0.698 |
| d.51.1 | 300 | 0.763 | 0.838 | 0.762 | 294 | 0.761 | 0.829 |
| d.54.1 | 351 | 0.804 | 0.854 | 0.815 | 349 | 0.808 | 0.855 |
| d.58.1 | 376 | 0.731 | 0.590 | 0.740 | 291 | 0.737 | 0.510 |
| d.58.18 | 406 | 0.618 | 0.726 | 0.588 | 395 | 0.624 | 0.722 |
| d.81.1 | 1176 | 0.748 | 0.877 | 0.755 | 530 | 0.803 | 0.874 |
| d.87.1 | 210 | 0.868 | 0.949 | 0.862 | 186 | 0.907 | 0.950 |
| d.104.1 | 528 | 0.697 | 0.848 | 0.719 | 225 | 0.697 | 0.855 |
| d.108.1 | 2775 | 0.665 | 0.734 | 0.663 | 1977 | 0.618 | 0.657 |
| d.113.1 | 496 | 0.603 | 0.690 | 0.611 | 326 | 0.588 | 0.678 |
| d.122.1 | 253 | 0.750 | 0.855 | 0.767 | 129 | 0.849 | 0.887 |
| d.131.1 | 276 | 0.799 | 0.804 | 0.806 | 233 | 0.777 | 0.772 |
| d.142.1 | 276 | 0.550 | 0.709 | 0.563 | 90 | 0.748 | 0.803 |
| d.144.1 | 2926 | 0.845 | 0.888 | 0.876 | 2314 | 0.723 | 0.741 |
| d.153.1 | 1225 | 0.865 | 0.941 | 0.872 | 722 | 0.827 | 0.825 |
| d.157.1 | 253 | 0.812 | 0.839 | 0.821 | 131 | 0.879 | 0.847 |
| d.159.1 | 253 | 0.859 | 0.921 | 0.862 | 60 | 0.959 | 0.934 |
| d.218.1 | 210 | 0.500 | 0.733 | 0.475 | 58 | 0.585 | 0.762 |
| e.8.1 | 666 | 0.706 | 0.876 | 0.726 | 78 | 0.868 | 0.794 |

^a^ The number of all the alignments within each superfamily.

^b^ Bilinear multiple correlation coefficient obtained by fitting Group 1 variables (PNI, SNG versus RMSD) for all the alignments within each superfamily, all 68 R are statistically significant (*p*<0.001).

^c^ Bilinear multiple correlation coefficient obtained by fitting Group 2 variables (PNS, LSNG versus Z-score) for all the alignments within each superfamily, all 68 R are statistically significant (*p*<0.001).

^d^ Bilinear multiple correlation coefficient obtained by fitting corrected Group 1 variables (PC, SNG versus RMSD) for all the alignments within each superfamily, all 68 R are statistically significant (*p*<0.001). PC is Poisson correction distance that is used to correct for multiple substitutions at the same site.

^e^ The number of the accurate alignments (P-score>0) within each superfamily.

^f^ Bilinear multiple correlation coefficient obtained by fitting Group 1 variables for the accurate alignments (P-score>0) within each superfamily, all 68 R are statistically significant (*p*<0.001).

^g^ Bilinear multiple correlation coefficient obtained by fitting Group 2 variables for the accurate alignments (P-score>0) within each superfamily, all 68 R are statistically significant (*p*<0.001).
