# Supplementary material for: Contributions of substitutions and indels to the structural variations in ancient protein superfamilies
Source: BMC Genomics. 2018 Oct 24;19:771. doi: 10.1186/s12864-018-5178-8 (PMC6201574; doi:10.1186/s12864-018-5178-8)
Supplement: Supplementary file 3 — Table S3. Results of adequacy analysis. (DOCX 22 kb) [file 12864_2018_5178_MOESM3_ESM.docx]

**Table S3. Results of adequacy analysis.**

| SCOP  code | Group 1^a^ | | | Group 2^b^ | | | adequacy3^f^ |
| --- | --- | --- | --- | --- | --- | --- | --- |
|  | **adjR^2 c^** | **adequacy1^d^** | **adequacy2^e^** | **adjR^2^** | **adequacy1** | **adequacy2** |  |
| a.4.5 | 0.360 | 2.559 | 1.175 | 0.462 | 2.180 | 1.135 | 1.281 |
| a.25.1 | 0.550 | 1.418 | 1.289 | 0.682 | 1.102 | 1.795 | 1.240 |
| a.35.1 | 0.390 | 1.547 | 1.447 | 0.550 | 1.509 | 1.356 | 1.413 |
| a.100.1 | 0.268 | 1.149 | 2.538 | 0.502 | 1.087 | 2.759 | 1.877 |
| b.36.1 | 0.447 | 3.292 | 1.031 | 0.484 | 2.710 | 1.023 | 1.083 |
| b.38.1 | 0.550 | 2.047 | 1.032 | 0.605 | 1.493 | 1.097 | 1.100 |
| b.40.4 | 0.246 | 1.634 | 1.504 | 0.374 | 1.378 | 1.634 | 1.519 |
| b.43.3 | 0.511 | 1.486 | 1.088 | 0.522 | 1.324 | 1.070 | 1.022 |
| b.45.1 | 0.521 | 1.579 | 1.282 | 0.541 | 1.173 | 1.551 | 1.038 |
| b.82.1 | 0.489 | 2.069 | 1.045 | 0.501 | 1.277 | 1.302 | 1.025 |
| b.92.1 | 0.707 | 1.459 | 1.047 | 0.599 | 1.252 | 1.036 | 0.847 |
| b.122.1 | 0.479 | 1.287 | 1.231 | 0.676 | 1.249 | 1.255 | 1.412 |
| c.1.2 | 0.680 | 1.212 | 1.123 | 0.750 | 1.192 | 1.112 | 1.103 |
| c.1.4 | 0.797 | 1.183 | 1.073 | 0.862 | 1.048 | 1.109 | 1.081 |
| c.1.9 | 0.708 | 1.167 | 1.144 | 0.813 | 1.142 | 1.118 | 1.149 |
| c.1.10 | 0.711 | 1.133 | 1.176 | 0.862 | 1.087 | 1.173 | 1.211 |
| c.1.11 | 0.772 | 1.210 | 1.126 | 0.851 | 1.094 | 1.171 | 1.102 |
| c.1.12 | 0.809 | 1.102 | 1.117 | 0.898 | 1.078 | 1.100 | 1.110 |
| c.2.1 | 0.466 | 1.269 | 1.364 | 0.578 | 1.267 | 1.306 | 1.241 |
| c.3.1 | 0.415 | 1.794 | 1.197 | 0.350 | 1.068 | 2.168 | 0.843 |
| c.14.1 | 0.624 | 1.391 | 1.103 | 0.726 | 1.210 | 1.178 | 1.164 |
| c.23.16 | 0.489 | 1.158 | 1.475 | 0.621 | 1.161 | 1.346 | 1.271 |
| c.26.1 | 0.550 | 1.142 | 1.263 | 0.735 | 1.129 | 1.202 | 1.338 |
| c.26.2 | 0.435 | 1.220 | 1.325 | 0.631 | 1.094 | 1.355 | 1.451 |
| c.31.1 | 0.498 | 1.096 | 1.427 | 0.644 | 1.254 | 1.250 | 1.293 |
| c.36.1 | 0.709 | 1.130 | 1.194 | 0.804 | 1.103 | 1.186 | 1.134 |
| c.37.1 | 0.394 | 1.145 | 1.651 | 0.575 | 1.175 | 1.511 | 1.459 |
| c.47.1 | 0.465 | 1.162 | 1.550 | 0.601 | 1.210 | 1.357 | 1.292 |
| c.52.1 | 0.214 | 1.402 | 1.580 | 0.482 | 1.280 | 1.766 | 2.249 |
| c.55.1 | 0.320 | 1.071 | 1.982 | 0.592 | 1.115 | 1.594 | 1.848 |
| c.55.3 | 0.318 | 1.129 | 1.597 | 0.529 | 1.112 | 1.550 | 1.664 |
| c.56.5 | 0.752 | 1.101 | 1.164 | 0.856 | 1.069 | 1.140 | 1.137 |
| c.58.1 | 0.783 | 1.116 | 1.139 | 0.884 | 1.064 | 1.166 | 1.128 |
| c.61.1 | 0.535 | 1.138 | 1.354 | 0.726 | 1.099 | 1.329 | 1.357 |
| c.66.1 | 0.473 | 1.491 | 1.241 | 0.615 | 1.392 | 1.237 | 1.301 |
| c.67.1 | 0.662 | 1.271 | 1.088 | 0.740 | 1.163 | 1.091 | 1.118 |
| c.68.1 | 0.606 | 1.264 | 1.180 | 0.770 | 1.107 | 1.311 | 1.270 |
| c.72.1 | 0.540 | 1.406 | 1.077 | 0.657 | 1.218 | 1.136 | 1.217 |
| c.78.1 | 0.810 | 1.136 | 1.083 | 0.866 | 1.087 | 1.047 | 1.069 |
| c.79.1 | 0.711 | 1.069 | 1.253 | 0.716 | 1.066 | 1.224 | 1.007 |
| c.87.1 | 0.630 | - | - | 0.821 | 1.074 | 1.144 | 1.302 |
| c.94.1 | 0.617 | 1.113 | 1.183 | 0.755 | 1.073 | 1.191 | 1.224 |
| c.95.1 | 0.603 | 1.227 | 1.115 | 0.773 | 1.155 | 1.126 | 1.282 |
| c.97.1 | 0.686 | 1.081 | 1.327 | 0.754 | 1.107 | 1.330 | 1.098 |
| c.108.1 | 0.438 | 1.597 | 1.180 | 0.589 | 1.545 | 1.202 | 1.346 |
| c.124.1 | 0.590 | 1.205 | 1.166 | 0.606 | 1.118 | 1.221 | 1.026 |
| d.14.1 | 0.253 | 1.135 | 1.870 | 0.497 | 1.255 | 1.584 | 1.963 |
| d.26.1 | 0.640 | 1.154 | 1.220 | 0.592 | 1.236 | 1.085 | 0.926 |
| d.37.1 | 0.228 | - | - | 0.324 | 1.675 | 1.746 | 1.422 |
| d.50.1 | 0.367 | 1.551 | 1.243 | 0.524 | 1.384 | 1.135 | 1.427 |
| d.51.1 | 0.580 | 1.891 | 1.107 | 0.700 | 1.154 | 1.305 | 1.207 |
| d.54.1 | 0.644 | 1.112 | 1.326 | 0.727 | 1.061 | 1.272 | 1.128 |
| d.58.1 | 0.532 | 1.209 | 1.459 | 0.345 | 1.239 | 1.350 | 0.648 |
| d.58.18 | 0.378 | 1.196 | 2.094 | 0.525 | 1.250 | 1.709 | 1.389 |
| d.81.1 | 0.558 | 1.106 | 1.290 | 0.769 | 1.063 | 1.379 | 1.377 |
| d.87.1 | 0.751 | 1.097 | 1.096 | 0.899 | 1.048 | 1.085 | 1.197 |
| d.104.1 | 0.485 | 1.292 | 1.177 | 0.719 | 1.211 | 1.238 | 1.483 |
| d.108.1 | 0.442 | 1.395 | 1.269 | 0.538 | 1.494 | 1.179 | 1.218 |
| d.113.1 | 0.361 | 1.662 | 1.158 | 0.474 | 1.531 | 1.181 | 1.313 |
| d.122.1 | 0.559 | 1.175 | 1.260 | 0.729 | 1.126 | 1.262 | 1.304 |
| d.131.1 | 0.635 | 1.603 | 1.049 | 0.644 | 1.348 | 1.077 | 1.014 |
| d.142.1 | 0.297 | 1.345 | 1.229 | 0.499 | 1.250 | 1.227 | 1.680 |
| d.144.1 | 0.714 | 1.301 | 1.057 | 0.789 | 1.164 | 1.055 | 1.105 |
| d.153.1 | 0.747 | 1.300 | 1.077 | 0.885 | 1.148 | 1.107 | 1.185 |
| d.157.1 | 0.657 | 1.204 | 1.199 | 0.701 | 1.231 | 1.146 | 1.068 |
| d.159.1 | 0.736 | 1.214 | 1.087 | 0.847 | 1.081 | 1.142 | 1.150 |
| d.218.1 | 0.243 | 1.569 | 1.192 | 0.533 | 1.281 | 1.359 | 2.192 |
| e.8.1 | 0.496 | 1.446 | 1.084 | 0.767 | 1.104 | 1.239 | 1.545 |

^a^ The results of adequacy analysis obtained by using Group 1 variables (PNI, SNG versus RMSD) for all alignments within each of 68 superfamilies.

^b^ The results of adequacy analysis obtained by using Group 2 variables (PNS, LSNG versus Z-score) for all alignments within each of 68 superfamilies.

^c^ The adjR^2^ is the coefficient of determination adjusted according to the number of independent variables and the number of data points in the fitting model.

^d^ The adequacy of the bilinear fitting versus linear fitting only considering substitutions within each superfamily. Due to not obtaining the statistically significant partial correlation coefficients within c.87.1 and d.37.1, the two superfamilies were excluded from the adequacy analysis.

^e^ The adequacy of the bilinear fitting versus linear fitting only considering indels within each superfamily.

^f^ The adequacy of the bilinear fitting produced by using Group 2 variables versus bilinear fitting obtained by adopting Group 1 variables within each superfamily.
