# Supplementary material for: Contributions of substitutions and indels to the structural variations in ancient protein superfamilies
Source: BMC Genomics. 2018 Oct 24;19:771. doi: 10.1186/s12864-018-5178-8 (PMC6201574; doi:10.1186/s12864-018-5178-8)
Supplement: Supplementary file 4 — Table S4. Fitting results of bilinear model within each of 68 ancient superfamilies. (DOCX 24 kb) [file 12864_2018_5178_MOESM4_ESM.docx]

**Table S4. Fitting results of bilinear model within each of 68 ancient superfamilies.**

| SCOP code | Group 1^a^ | | | | Group 2^b^ | | | |
| --- | --- | --- | --- | --- | --- | --- | --- | --- |
|  | **R** | **b_1_^c^** | **b_2_^d^** | **VIF^e^** | **R** | **b_1_^f^** | **b_2_^g^** | **VIF** |
| a.4.5 | 0.600 | 0.024 | 0.082 | 1.082 | 0.679 | -0.044 | -0.148 | 1.170 |
| a.25.1 | 0.742 | 0.031 | 0.165 | 1.302 | 0.826 | -0.094 | -0.184 | 1.347 |
| a.35.1 | 0.625 | 0.018 | 0.073 | 1.125 | 0.743 | -0.045 | -0.130 | 1.189 |
| a.100.1 | 0.523 | 0.049 | 0.050 | 1.079 | 0.711 | -0.096 | -0.066 | 1.128 |
| b.36.1 | 0.669 | 0.007 | 0.072 | 1.186 | 0.696 | -0.023 | -0.220 | 1.297 |
| b.38.1 | 0.744 | 0.006 | 0.079 | 1.418 | 0.780 | -0.036 | -0.178 | 1.563 |
| b.40.4 | 0.496 | 0.029 | 0.051 | 1.082 | 0.612 | -0.063 | -0.085 | 1.130 |
| b.43.3 | 0.717 | 0.018 | 0.080 | 1.590 | 0.725 | -0.045 | -0.177 | 1.957 |
| b.45.1 | 0.724 | 0.022 | 0.073 | 1.204 | 0.737 | -0.111 | -0.172 | 1.343 |
| b.82.1 | 0.700 | 0.014 | 0.098 | 1.388 | 0.708 | -0.103 | -0.185 | 1.432 |
| b.92.1 | 0.843 | 0.020 | 0.134 | 1.868 | 0.776 | -0.048 | -0.233 | 2.558 |
| b.122.1 | 0.693 | 0.033 | 0.065 | 1.516 | 0.823 | -0.095 | -0.156 | 1.547 |
| c.1.2 | 0.825 | 0.017 | 0.074 | 2.057 | 0.867 | -0.094 | -0.305 | 2.208 |
| c.1.4 | 0.894 | 0.022 | 0.099 | 2.578 | 0.929 | -0.181 | -0.292 | 3.832 |
| c.1.9 | 0.842 | 0.032 | 0.086 | 2.135 | 0.902 | -0.145 | -0.327 | 2.452 |
| c.1.10 | 0.844 | 0.028 | 0.069 | 2.162 | 0.928 | -0.163 | -0.258 | 2.524 |
| c.1.11 | 0.880 | 0.018 | 0.076 | 2.041 | 0.923 | -0.129 | -0.266 | 2.455 |
| c.1.12 | 0.900 | 0.025 | 0.086 | 2.774 | 0.948 | -0.133 | -0.335 | 3.322 |
| c.2.1 | 0.683 | 0.038 | 0.061 | 1.375 | 0.760 | -0.130 | -0.201 | 1.446 |
| c.3.1 | 0.644 | 0.020 | 0.071 | 1.203 | 0.592 | -0.109 | -0.069 | 1.284 |
| c.14.1 | 0.790 | 0.016 | 0.066 | 1.704 | 0.853 | -0.117 | -0.242 | 1.835 |
| c.23.16 | 0.700 | 0.039 | 0.062 | 1.432 | 0.789 | -0.134 | -0.193 | 1.585 |
| c.26.1 | 0.742 | 0.034 | 0.055 | 1.809 | 0.858 | -0.120 | -0.194 | 2.067 |
| c.26.2 | 0.661 | 0.027 | 0.067 | 1.474 | 0.795 | -0.130 | -0.176 | 1.800 |
| c.31.1 | 0.708 | 0.030 | 0.041 | 1.641 | 0.804 | -0.108 | -0.207 | 1.539 |
| c.36.1 | 0.842 | 0.029 | 0.060 | 2.092 | 0.897 | -0.121 | -0.206 | 2.308 |
| c.37.1 | 0.628 | 0.053 | 0.053 | 1.340 | 0.758 | -0.127 | -0.134 | 1.383 |
| c.47.1 | 0.682 | 0.038 | 0.047 | 1.376 | 0.775 | -0.095 | -0.137 | 1.473 |
| c.52.1 | 0.465 | 0.031 | 0.055 | 1.126 | 0.695 | -0.091 | -0.106 | 1.143 |
| c.55.1 | 0.566 | 0.032 | 0.028 | 1.339 | 0.770 | -0.114 | -0.116 | 1.441 |
| c.55.3 | 0.564 | 0.028 | 0.050 | 1.399 | 0.728 | -0.098 | -0.119 | 1.485 |
| c.56.5 | 0.868 | 0.033 | 0.081 | 2.443 | 0.925 | -0.162 | -0.269 | 2.996 |
| c.58.1 | 0.886 | 0.032 | 0.083 | 2.460 | 0.940 | -0.108 | -0.168 | 2.819 |
| c.61.1 | 0.732 | 0.027 | 0.059 | 1.639 | 0.852 | -0.123 | -0.192 | 1.846 |
| c.66.1 | 0.688 | 0.030 | 0.070 | 1.302 | 0.784 | -0.117 | -0.218 | 1.389 |
| c.67.1 | 0.814 | 0.026 | 0.096 | 2.072 | 0.860 | -0.139 | -0.351 | 2.548 |
| c.68.1 | 0.779 | 0.021 | 0.062 | 1.685 | 0.878 | -0.136 | -0.167 | 1.848 |
| c.72.1 | 0.737 | 0.015 | 0.078 | 1.764 | 0.812 | -0.106 | -0.286 | 1.951 |
| c.78.1 | 0.901 | 0.027 | 0.128 | 2.798 | 0.931 | -0.078 | -0.295 | 4.253 |
| c.79.1 | 0.845 | 0.023 | 0.054 | 2.235 | 0.848 | -0.110 | -0.210 | 2.385 |
| c.87.1 | 0.796 | - | 0.174 | 2.397 | 0.907 | -0.186 | -0.321 | 2.847 |
| c.94.1 | 0.786 | 0.032 | 0.078 | 2.249 | 0.869 | -0.131 | -0.193 | 2.553 |
| c.95.1 | 0.777 | 0.026 | 0.108 | 2.046 | 0.879 | -0.107 | -0.293 | 2.315 |
| c.97.1 | 0.830 | 0.030 | 0.056 | 1.916 | 0.869 | -0.104 | -0.178 | 1.788 |
| c.108.1 | 0.662 | 0.025 | 0.072 | 1.311 | 0.768 | -0.102 | -0.220 | 1.314 |
| c.124.1 | 0.770 | 0.025 | 0.068 | 1.864 | 0.780 | -0.103 | -0.154 | 2.020 |
| d.14.1 | 0.505 | 0.034 | 0.040 | 1.243 | 0.706 | -0.084 | -0.133 | 1.230 |
| d.26.1 | 0.801 | 0.020 | 0.060 | 1.875 | 0.771 | -0.056 | -0.212 | 2.175 |
| d.37.1 | 0.483 | - | 0.056 | 1.001 | 0.573 | -0.048 | -0.087 | 1.024 |
| d.50.1 | 0.610 | 0.014 | 0.068 | 1.239 | 0.726 | -0.040 | -0.166 | 1.578 |
| d.51.1 | 0.763 | 0.016 | 0.096 | 1.281 | 0.838 | -0.076 | -0.142 | 1.670 |
| d.54.1 | 0.804 | 0.025 | 0.073 | 1.774 | 0.854 | -0.081 | -0.149 | 2.256 |
| d.58.1 | 0.731 | 0.029 | 0.049 | 1.356 | 0.590 | -0.055 | -0.085 | 1.398 |
| d.58.18 | 0.618 | 0.034 | 0.038 | 1.120 | 0.726 | -0.069 | -0.098 | 1.179 |
| d.81.1 | 0.748 | 0.035 | 0.083 | 1.898 | 0.877 | -0.128 | -0.164 | 1.948 |
| d.87.1 | 0.868 | 0.021 | 0.063 | 3.026 | 0.949 | -0.090 | -0.173 | 4.284 |
| d.104.1 | 0.697 | 0.019 | 0.073 | 1.623 | 0.848 | -0.122 | -0.291 | 1.661 |
| d.108.1 | 0.665 | 0.028 | 0.064 | 1.342 | 0.734 | -0.073 | -0.205 | 1.388 |
| d.113.1 | 0.603 | 0.022 | 0.072 | 1.293 | 0.690 | -0.073 | -0.178 | 1.345 |
| d.122.1 | 0.750 | 0.019 | 0.066 | 1.680 | 0.855 | -0.104 | -0.214 | 1.867 |
| d.131.1 | 0.799 | 0.017 | 0.104 | 1.656 | 0.804 | -0.082 | -0.292 | 1.891 |
| d.142.1 | 0.550 | 0.022 | 0.057 | 1.397 | 0.709 | -0.090 | -0.167 | 1.572 |
| d.144.1 | 0.845 | 0.020 | 0.115 | 2.212 | 0.888 | -0.089 | -0.326 | 2.990 |
| d.153.1 | 0.865 | 0.025 | 0.096 | 2.051 | 0.941 | -0.131 | -0.267 | 2.506 |
| d.157.1 | 0.812 | 0.036 | 0.066 | 1.763 | 0.839 | -0.139 | -0.267 | 1.868 |
| d.159.1 | 0.859 | 0.022 | 0.086 | 2.260 | 0.921 | -0.155 | -0.250 | 2.792 |
| d.218.1 | 0.500 | 0.015 | 0.050 | 1.246 | 0.733 | -0.087 | -0.145 | 1.341 |
| e.8.1 | 0.706 | 0.018 | 0.113 | 1.690 | 0.876 | -0.128 | -0.193 | 2.064 |

^a^ The results obtained by using bilinear model to fit Group 1 variables (PNI, SNG versus RMSD) for all the alignments within each superfamily.

^b^ The results obtained by using bilinear model to fit Group 2 variables (PNS, LSNG versus Z-score) for all the alignments within each superfamily.

^c^ The regression coefficient b_1_ obtained by fitting Group 1 variables. It is termed as structural substitution sensitivity (SSS). The statistical significance of both b_1_ and the partial correlation coefficient r_x1y·x2_ are equal with each other. Except for c.87.1 (*p*=0.038) and d.37.1 (*p*=0.236), all the b_1_ of other superfamilies are significantly different from zero (*p*<0.01).

^d^ The regression coefficient b_2_ obtained by fitting Group 1 variables. It is termed as structural indel sensitivity (SIDS). The statistical significance of b_2_ is equal to that of the partial correlation coefficient r_x2y·x1_. The b_2_ of all the superfamilies are significantly different from zero (*p*<0.001).

^e^ Variance inflation factor (VIF). It quantifies the severity of multicollinearity. A common rule of thumb is that if VIF for one of the variables is greater than 5, there is collinearity associated with that variable.

^f^ The regression coefficient b_1_ obtained by fitting Group 2 variables. It is termed as SSS. They are significantly different from zero (*p*<0.001) for all the superfamilies.

^g^ The regression coefficient b_2_ obtained by fitting Group 2 variables. It is termed as SIDS. They are significantly different from zero (*p*<0.001) for all the superfamilies.
