# Supplementary material for: Contributions of substitutions and indels to the structural variations in ancient protein superfamilies
Source: BMC Genomics. 2018 Oct 24;19:771. doi: 10.1186/s12864-018-5178-8 (PMC6201574; doi:10.1186/s12864-018-5178-8)
Supplement: Supplementary file 5 — Table S5. Accumulated contributions of substitutions and indels to structural variations within the ancient superfamilies. (DOCX 17 kb) [file 12864_2018_5178_MOESM5_ESM.docx]

**Table S5. Accumulated contributions of substitutions and indels to structural variations within the ancient superfamilies.**

| SCOP code | R^a^ | Contribution 1^b^ | Contribution 2^c^ | C1/C2^d^ |
| --- | --- | --- | --- | --- |
| a.25.1 | 0.826 | 0.502 | 0.180 | 2.781 |
| b.38.1 | 0.780 | 0.190 | 0.418 | 0.454 |
| b.92.1 | 0.776 | 0.174 | 0.429 | 0.404 |
| b.122.1 | 0.823 | 0.341 | 0.336 | 1.013 |
| c.1.2 | 0.867 | 0.325 | 0.426 | 0.764 |
| c.1.4 | 0.929 | 0.518 | 0.345 | 1.503 |
| c.1.9 | 0.902 | 0.388 | 0.425 | 0.913 |
| c.1.10 | 0.928 | 0.504 | 0.357 | 1.412 |
| c.1.11 | 0.923 | 0.488 | 0.363 | 1.345 |
| c.1.12 | 0.948 | 0.477 | 0.422 | 1.129 |
| c.2.1 | 0.760 | 0.299 | 0.279 | 1.072 |
| c.14.1 | 0.853 | 0.348 | 0.378 | 0.921 |
| c.23.16 | 0.789 | 0.369 | 0.253 | 1.461 |
| c.26.1 | 0.858 | 0.409 | 0.327 | 1.249 |
| c.26.2 | 0.795 | 0.416 | 0.216 | 1.923 |
| c.31.1 | 0.804 | 0.322 | 0.324 | 0.991 |
| c.36.1 | 0.897 | 0.461 | 0.344 | 1.341 |
| c.37.1 | 0.758 | 0.362 | 0.212 | 1.709 |
| c.47.1 | 0.775 | 0.340 | 0.261 | 1.303 |
| c.55.1 | 0.770 | 0.411 | 0.181 | 2.269 |
| c.56.5 | 0.925 | 0.503 | 0.353 | 1.423 |
| c.58.1 | 0.940 | 0.544 | 0.341 | 1.597 |
| c.61.1 | 0.852 | 0.469 | 0.258 | 1.822 |
| c.66.1 | 0.784 | 0.269 | 0.346 | 0.778 |
| c.67.1 | 0.860 | 0.316 | 0.424 | 0.747 |
| c.68.1 | 0.878 | 0.485 | 0.285 | 1.702 |
| c.72.1 | 0.812 | 0.292 | 0.368 | 0.793 |
| c.78.1 | 0.931 | 0.368 | 0.500 | 0.735 |
| c.79.1 | 0.848 | 0.463 | 0.256 | 1.809 |
| c.87.1 | 0.907 | 0.478 | 0.345 | 1.388 |
| c.94.1 | 0.869 | 0.467 | 0.289 | 1.617 |
| c.95.1 | 0.879 | 0.366 | 0.407 | 0.901 |
| c.97.1 | 0.869 | 0.480 | 0.276 | 1.741 |
| c.108.1 | 0.768 | 0.223 | 0.366 | 0.610 |
| c.124.1 | 0.780 | 0.351 | 0.258 | 1.360 |
| d.26.1 | 0.771 | 0.224 | 0.370 | 0.607 |
| d.51.1 | 0.838 | 0.410 | 0.293 | 1.400 |
| d.54.1 | 0.854 | 0.493 | 0.236 | 2.089 |
| d.81.1 | 0.877 | 0.546 | 0.223 | 2.448 |
| d.87.1 | 0.949 | 0.512 | 0.388 | 1.317 |
| d.104.1 | 0.848 | 0.370 | 0.349 | 1.061 |
| d.122.1 | 0.855 | 0.431 | 0.300 | 1.435 |
| d.131.1 | 0.804 | 0.210 | 0.437 | 0.480 |
| d.144.1 | 0.888 | 0.290 | 0.499 | 0.580 |
| d.153.1 | 0.941 | 0.407 | 0.478 | 0.851 |
| d.157.1 | 0.839 | 0.312 | 0.391 | 0.800 |
| d.159.1 | 0.921 | 0.482 | 0.366 | 1.319 |
| e.8.1 | 0.876 | 0.462 | 0.306 | 1.510 |

^a^ The superfamilies with bilinear multiple correlation greater than 0.75, the R was obtained by using Group 2 (PNS, LSNG versus Z-score) to fit all the alignments within each superfamily.

^b^ The accumulated contributions of substitutions to structural variations within each superfamily.

^c^ The accumulated contributions of indels to structural variations within each superfamily.

^d^ The ratio of the contributions of substitutions versus those of indels within each superfamily.
